# Vitamin D Status Determines the Impact of Metformin on Gonadotropin Levels in Postmenopausal Women

**DOI:** 10.3390/jcm12113715

**Published:** 2023-05-27

**Authors:** Robert Krysiak, Karolina Kowalcze, Witold Szkróbka, Bogusław Okopień

**Affiliations:** 1 Department of Internal Medicine and Clinical Pharmacology, Medical University of Silesia, Medyków 18, 40-752 Katowice, Poland; wszkrobka@sum.edu.pl (W.S.); bokopien@sum.edu.pl (B.O.); 2 Department of Pediatrics in Bytom, School of Health Sciences in Katowice, Medical University of Silesia, Stefana Batorego 15, 41-902 Bytom, Poland

**Keywords:** insulin resistance, gonadotrope secretory function, menopause, vitamin D

## Abstract

Metformin was found to decrease elevated levels of anterior pituitary hormones. Its impact on lactotrope secretory function was absent in women with vitamin D insufficiency. This study investigated whether vitamin D status determines metformin action on overactive gonadotropes. We compared the effect of six-month metformin treatment on the plasma levels of gonadotropins, TSH, prolactin, ACTH, estradiol, free thyroid hormones, IGF-1, and 25-hydroxyvitamin D, as well as on glucose homeostasis markers between three matched groups of postmenopausal women at high risk for diabetes: untreated subjects with vitamin D insufficiency (group A), untreated women with normal vitamin D status (group B), and individuals receiving vitamin D supplementation with normal 25-hydroxyvitamin D levels (group C). Only in groups B and C did metformin reduce FSH levels and tend to decrease LH levels, and these effects correlated with baseline gonadotropin levels, baseline 25-hydroxyvitamin D levels, and the improvement in insulin sensitivity. Follow-up gonadotropin levels were higher in group A than in the other two groups. The drug did not affect circulating levels of TSH, prolactin, ACTH, estradiol, free thyroid hormones, IGF-1, or 25-hydroxyvitamin D. The obtained results suggest that the impact of metformin on gonadotropin secretion in women after menopause is determined by vitamin D status.

## 1. Introduction

In recent years, it has become clear that metformin, the main first-line medication for the treatment of type 2 diabetes and other insulin resistance states [1], affects the secretory function of the anterior lobe of the pituitary gland [2,3,4,5,6,7,8,9,10]. The drug was found to reduce circulating levels of thyroid-stimulating hormone (TSH) [2,3], prolactin [4,5], and gonadotropins [6,7,8,9,10] if baseline levels of these hormones were elevated. Metformin decreased follicle-stimulating hormone (FSH) levels in postmenopausal women [6,7] and in men with hypogonadism [8], as well as luteinizing hormone (LH) levels in women with polycystic ovary syndrome [9,10] and in men with primary testicular failure [8]. Its gonadotropin-lowering properties were potentiated by rosuvastatin treatment [11]. The inhibitory effect of metformin on the secretion of anterior pituitary hormones seems to result from its action at the level of anterior pituitary cells and is attributed to the lack of the blood–brain barrier in the pituitary gland [12]. It has been found that oral administration of metformin, both in the short and long term, resulted in higher tissue levels of this agent in the pituitary than in the remaining assessed brain regions: the hypothalamus, frontal cortex, cerebellum, hippocampus, striatum, and olfactory bulbs [13]. In primary rat pituitary cells, metformin inhibited in a dose-dependent manner the gonadotropin-releasing hormone-induced secretion of FSH and LH and the activin-induced secretion of FSH [14]. This dose dependence was also observed in clinical studies. Only high-dose (2.55–3 g), not low-dose (1.7 g), metformin treatment decreased FSH levels [6], and similar relationships were observed in its impact on prolactin levels [5]. These findings suggest that pituitary cells are less sensitive to metformin than other target tissues (the liver, skeletal muscle, and adipose tissues), and high quantities of this agent in the pituitary gland are required to inhibit its secretory function. The impact of metformin on the secretion of pituitary hormones is postulated to be mediated by 5′-adenosine monophosphate-activated protein kinase (AMPK), a major cellular regulator of lipid and glucose metabolism [15] and a mediator of many biological effects of metformin [16]. Interestingly, gonadotropes are pituitary cells with the highest expression of this enzyme [14]. Lastly, metformin shows anti-tumor and anti-inflammatory properties associated with suppressing the NF-κB signaling pathway [17].

Menopause significantly increases the risk of cardiometabolic diseases, such as obesity, type 2 diabetes, cardiovascular diseases, non-alcoholic liver disease, and metabolic syndrome [18]. Most cardiometabolic disorders, including obesity, type 2 diabetes, metabolic syndrome, and polycystic ovary syndrome, are characterized by an increased prevalence of hypovitaminosis D and lower levels of 25-hydroxyvitamin D [19,20]. Vitamin D (calciferol) supplementation has a favorable impact on insulin sensitivity, hyperglycemia, dyslipidemia, obesity, and hypertension [21]. Low vitamin D status is a frequent finding in postmenopausal women [22]. This means that many postmenopausal women with type 2 diabetes, metabolic syndrome, and other insulin resistance states are candidates for metformin/vitamin D combination therapy. In a randomized controlled trial, metformin administered to subjects with type 2 diabetes and low vitamin D status together with exogenous calciferol was superior to metformin administered alone in reducing whole blood levels of glycated hemoglobin (HbA_1c_) [23]. The beneficial effect of metformin/vitamin D combination therapy may be associated with the stimulation of the AMPK pathway. Metformin has been shown to exert many metabolic effects via activation of this kinase [16], and a similar effect on its activity was observed in animals supplemented with vitamin D [24]. Moreover, the synergistic effects of metformin and calcitriol (the active form of vitamin D) on the proliferation and apoptosis of human prostate cancer cells were mediated by the AMK pathway [25].

Recently, we have reported that metformin and vitamin D may interact at the level of pituitary cells. Metformin reduced circulating prolactin concentration only in subjects with 25-hydroxyvitamin D levels within the reference range [26]. Moreover, in women with vitamin D insufficiency, the TSH-lowering effect of metformin was observed only if this drug was administered together with exogenous calciferol but not if it was administered alone [27]. To the best of our knowledge, no previous study has assessed the interactions between metformin and vitamin D on gonadotropin secretion. To fill in this gap, the aim of the present study was to investigate whether vitamin D status determines the impact of metformin on hypothalamic–pituitary–ovarian axis activity in postmenopausal women.

## 2. Materials and Methods

This study was conducted in accordance with the principles of the Declaration of Helsinki, and all participants provided written informed consent prior to enrollment. The study protocol was approved by the institutional review board. Because the study did not meet the criteria of a clinical trial (the patients were not randomized and received the same drug), registration in a public trials registry before the time of first patient enrollment was not applicable. The manuscript was prepared in accordance with the Quality and Transparency of Health Research (EQUATOR) Network guidelines for observational studies (STROBE).

### 2.1. Patients

**Figure 1:**
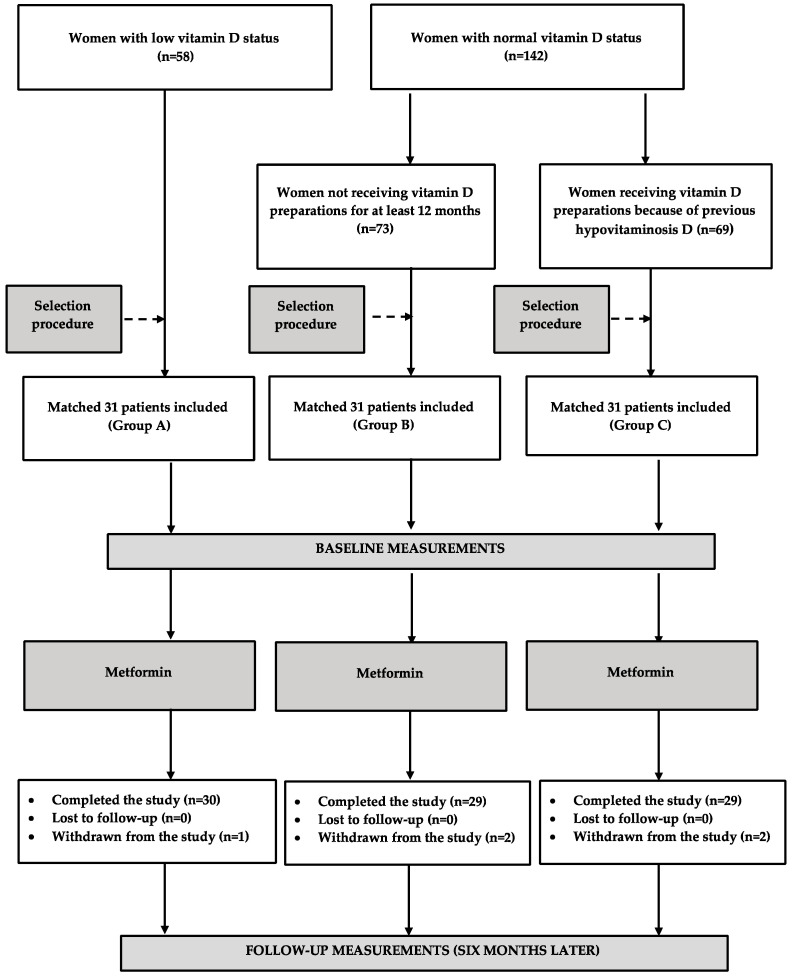
The flow chart of patients through the study.

The study population consisted of 3 groups of postmenopausal women (aged 50–70 years) at high risk for diabetes. Postmenopause was defined as no periods for over 12 months, coexisting with high plasma levels of FSH (>30 IU/L) and low plasma levels of estradiol (<30 pg/mL), found on 2 different occasions. Women were considered eligible for enrollment if they met all the following criteria: body mass index (BMI) of at least 24 kg/m^2^, 2 h post-challenge plasma glucose between 140 and 200 mg/dL (impaired glucose tolerance), and fasting plasma glucose in the range between 95 and 125 mg/dL, despite complying for at least 3 months with the lifestyle modification program. The same criteria defining patients at high risk for diabetes were used in the Diabetes Prevention Program, the largest and longest clinical trial for the prevention of diabetes conducted to date [28]. Group A included women with vitamin D insufficiency, defined as plasma 25-hydroxyvitamin D levels between 20 and 30 ng/mL. Groups B and C included women with normal vitamin D status, defined as 25-hydroxyvitamin D levels between 30 and 60 ng/mL. Patients assigned to group B had not received vitamin D preparations for at least 12 months before enrollment. Group C consisted of women receiving vitamin D preparations (4000 IU (100 μg) daily) because of previous hypovitaminosis D. The participants (31 women in each group) were selected from a larger number of eligible candidates based on a computer algorithm (Figure 1) aimed at creating 3 study groups matched for age, glucose homeostasis markers, and blood pressure. The number of patients exceeded the required number. An a priori sample size calculation showed that at least 28 participants were needed in each group to detect a 20% between-group difference in FSH levels (the primary endpoint) with 80% power and α error of 0.05. The 20% threshold in the primary endpoint seems to be the minimal clinically relevant and biologically plausible difference between the studied groups [6,11]. The expected probability of the primary endpoint was estimated based on the results of our previous studies [6,7]. To minimize the possible impact of seasonal fluctuations in the outcome variables [29,30,31], 44 women (15 in group A, 15 in group B, and 14 in group C) were recruited between January and February and the remaining 44 women in July or August.

The exclusion criteria were as follows: 25-hydroxyvitamin D levels <20 ng/mL, diabetes, other endocrine disorders, chronic inflammatory or autoimmune disorders, cardiovascular disease (except for mild arterial hypertension), kidney or liver failure, malabsorption syndromes, other serious disorders, pregnancy or lactation, any treatment (except for exogenous vitamin D in group C), and poor patient compliance.

### 2.2. Study Design

All participants of this prospective matched cohort study were treated for six months with oral metformin. In the first week, they received 500 mg of this agent twice a day. For the following 2 weeks, metformin dose was increased to 0.85–1 g twice a day. From week 4 onward, the daily dose of metformin was 2.55–3 g, and the drug was administered in 3 equal doses. In order to improve metformin tolerance, tablets were taken with or immediately after meals. The dose of vitamin D in group B was the same as before enrollment. Short-term (below seven days) use of new drugs (non-steroidal anti-inflammatory drugs, acetaminophen, other pain relievers, loperamide, antiemetic drugs, laxatives, or zolipidem) was accepted only if such treatment was terminated at least six weeks before final measurements. The participants were also asked to further comply with the lifestyle modification program. Medication adherence was measured every six weeks by counting the number of returned tables and analysis of responses in the Morisky, Green, and Levine Medication Adherence Scale [32], while compliance with non-pharmacological recommendations was assessed by analysis of individual eating diaries. They were regarded as “adherent” if the percentage of tablets returned was in the range from 0% to 10% and the overall score in the Morisky, Green, and Levine Medication Adherence Scale was 0 [32].

### 2.3. Laboratory Assays

Venous blood samples were collected between 8.00 and 8.30 a.m. after 12 h overnight fasting in a quiet and air-conditioned room (constant temperature of 23–24 °C) on the first and last study day. To minimize analytical errors, all assays were carried out in duplicate according to manufacturers’ instructions, and final results were averaged. A technician carrying out laboratory assays was unaware of patients’ personal data, clinical status, treatment group, and study sequence. Fasting plasma glucose was assessed by the hexokinase method with the use of a biochemical analyzer (Roche Cobas C311, Mannheim, Germany). Whole blood glycated hemoglobin (HbA_1c_) was measured using turbidimetric inhibition immunoassay on the Cobas Integra 800 analyzer (Roche Diagnostics, Mannheim, Germany). Plasma concentrations of insulin, gonadotropins (FSH and LH), TSH, prolactin, estradiol, free thyroid hormones (free thyroxine and free triiodothyronine), 25-hydroxyvitamin D, and anti-Müllerian hormone (AMH) were assayed by direct chemiluminescence using acridinium ester technology (ADVIA Centaur XP Immunoassay System, Siemens Healthcare Diagnostics, Munich, Germany). AMH was assessed only in samples of 10 patients from each group. Levels of adrenocorticotropic hormone (ACTH) and insulin-like growth factor-1 (IGF-1) were assessed by solid-phase enzyme-labeled chemiluminescent immunometric assays (Immulite, Siemens, Munich, Germany). Product codes were as follows: 04404483 (glucose), 05336180 (HbA_1c_), 2230141 (insulin), 1360521 (FSH), 2212941 (LH), 6491080 (TSH), 9505871 (prolactin), 10491445 (estradiol), 6490106 (free thyroxine), 3154228 (free triiodothyronine), 10631201 (25-hydroxyvitamin D), 10998432 (AMH), 10387014 (ACTH), 11128584 (IGF-1). The homeostatic model assessment 1 of insulin resistance ratio (HOMA1-IR) was calculated by multiplying plasma glucose (in mg/dL) by plasma insulin (in mU/L) and dividing by 405.

### 2.4. Statistical Analysis

To minimize heteroscedasticity, all data were subjected to log transformation before statistical analysis. The study groups and percentage changes from baseline were compared using one-way ANOVA followed by Bonferroni’s post hoc multiple comparison test. Comparisons between the subgroups were made with t-test for independent samples. Age, BMI, smoking, and blood pressure were considered as potential confounders. Baseline and follow-up values within the same treatment group were compared using Student’s paired *t*-test. All nominal data were compared using the chi-square test. Pearson’s r-tests were used to analyze the significance of correlations between the assessed variables. Statistical significance was defined as a two-tailed *p*-value of less than 0.05. All statistical analyses were performed using the Statistica 12.0 PL software package (number: JPZP507D199115ARCN-E, StatSoft Polska, Kraków, Poland).

## 3. Results

There were no differences between the study groups in age, smoking habits, BMI, blood pressure (both systolic and diastolic), glucose homeostasis markers (glucose, insulin, HOMA1-IR, and HbA_1c_), and the plasma levels of gonadotropins, TSH, prolactin, ACTH, estradiol, free thyroid hormones, IGF-1, and AMH. Expectedly, 25-hydroxyvitamin D levels were lowest in group A but did not differ between groups B and C (Table 1 and Table 2).

Two patients from group C were withdrawn because of loss of appetite and a general feeling of discomfort. Three other patients (one from group A and two from group B) stopped participating in the study because of nausea and vomiting. The remaining 88 patients (95%) completed the study, and their data were statistically analyzed. All these subjects complied with the treatment and dietary recommendations. A post hoc power calculation based on the primary outcome data and the given sample size showed that the study had sufficient statistical power. There were no between-group differences in the daily dose of metformin (group A: 2.78 ± 0.23 g; group B: 2.82 ± 0.21 g; group C: 2.80 ± 0.20 g) or in the daily intake of calciferol (not counting vitamin D tablets) (group A: 395 ± 180 IU; group B: 428 ± 173 IU; group C: 419 ± 195 IU).

Metformin decreased glucose, insulin, HOMA1-IR, and HbA_1c_ in all study groups. In groups B and C, the drug decreased FSH levels and tended to reduce LH levels, while in group A there were no differences between the baseline and follow-up concentrations of both gonadotropins. Circulating levels of 25-hydroxyvitamin D, TSH, prolactin, ACTH, estradiol, free thyroid hormones, IGF-1, and AMH remained at a similar level throughout the study period (Table 2). Groups B and C differed from group A in the percentage changes from baseline in glucose, insulin, HOMA1-IR, HbA_1c_, FSH, and LH (Table 3) and in the follow-up values of glucose, insulin, HOMA1-IR, HbA_1c_, 25-hydroxyvitamin D, FSH, and LH (Table 2). In all study groups, BMI and blood pressure did not differ significantly from their baseline values. The impact of metformin on the investigated variables did not differ between patients recruited in the winter and summer months (Table 4).

Metformin-induced changes in gonadotropin levels correlated with their baseline values (group A: FSH—r = 0.50 (*p* < 0.0001), LH—r = 0.37 (*p* = 0.0021); group B: FSH—r = 0.60 (*p* < 0.0001), LH—r = 0.42 (*p* = 0.0008); group C: FSH—r = 0.58 (*p* < 0.0001), LH—r = 0.43 (*p* = 0.0006)); concentrations of 25-hydroxyvitamin D (group A: FSH—r = 0.53 (*p* < 0.0001), LH—r = 0.46 (*p* = 0.0002); group B: FSH—r = 0.56 (*p* < 0.0001), LH—r = 0.39 (*p* = 0.0012); group C: FSH—r = 0.55 (*p* < 0.0001), LH—r = 0.41 (*p* = 0.0010)); metformin-induced decrease in insulin levels (group B: FSH—r = 0.37 (*p* = 0.0084), LH—r = 0.32 (*p* = 0.0354); group C: FSH—r = 0.35 (*p* = 0.0121), LH—r = 0.29 (*p* = 0.0482)); and metformin-induced decrease in HOMA1-IR (group A: FSH—r = 0.42 (*p* = 0.0006), LH—r = 0.32 (*p* = 0.0226); group B: FSH—r = 0.39 (*p* = 0.0010), LH—r = 0.34 (*p* = 0.0226); group C: FSH—r = 0.41 (*p* = 0.0006), LH—r = 0.31 (*p* = 0.0406)). The remaining correlations did not reach statistical significance.

## 4. Discussion

Although metformin administered to postmenopausal women with normal vitamin D status reduced circulating levels of both gonadotropins, its impact on FSH was more pronounced than on LH. Despite a reduction, post-treatment levels were still higher than in women of reproductive age. This difference in the action on both gonadotropins may be explained by the higher baseline levels of FSH than LH characterizing women after menopause. In line with this explanation, treatment-induced changes in FSH and LH levels correlated with their baseline values. Moreover, the drug had a neutral effect on circulating levels of the other anterior pituitary hormones (TSH, prolactin, and ACTH) and effector hormones (free thyroxine, free triiodothyronine, and IGF-1), concentrations of which were within normal limits. The novel finding of our study was that gonadotropin-lowering effects were absent in women with low vitamin D status.

The clinical significance of elevated gonadotropin levels in postmenopausal women is a matter of debate. However, more and more studies suggest their deteriorating effect on women’s health. FSH was found to increase bone resorption, promote weight gain, inhibit thermogenesis, and increase hepatic cholesterol production, and all these effects were absent in animals receiving antibodies against FSH [33,34]. Thus, elevated levels of this hormone may play an important role in the pathogenesis of bone loss, impaired energy homeostasis, and dyslipidemia, frequently observed in women after menopause [35]. In turn, high LH concentrations were found to correlate with cognitive deficits and incidence of Alzheimer’s disease, while pharmacological intervention directed at reducing circulating LH levels improved learning and memory in ovariectomized rodents and in animal models of Alzheimer’s disease [36,37]. These findings suggest that postmenopausal women may benefit from treatment with agents reducing gonadotropin levels, particularly with drugs decreasing both FSH and LH. Based on these observations, our findings allow us to draw some practical conclusions. Firstly, the benefits resulting from metformin treatment are not limited to metabolic effects. A statistically significant reduction in FSH coexisting with a small decrease in LH may prevent or delay unfavorable changes associated with aging. Despite a similar action on gonadotrope function, metformin seems to be a safer drug in comparison with hormone replacement therapy. The latter may be associated with an increased risk of cardiovascular disease and breast cancer [38], while metformin was shown to decrease the incidence of cardiovascular disease in patients with diabetes and to inhibit the growth of breast cancer [39]. To reduce gonadotropin levels, patients have to receive high doses of metformin, commonly used in type 2 diabetes but higher than recommended to prediabetic women [40]. However, chronic treatment with 2.55–3 g metformin daily was well tolerated by the participants of the current study, and there were no cases of serious adverse effects associated with high-dose metformin treatment in our previous studies [26,27]. Moreover, a network meta-analysis showed that 6-month treatment of overweight and obese subjects without diabetes with 3 g metformin daily was well tolerated and superior to treatment with lower doses in reducing body weight and improving glucose homeostasis [28]. Secondly, correlations with baseline levels suggest that metformin improves gonadotrope secretory function but does not seem to lead to gonadotrope hypofunction, the clinical consequences of which after menopause remain unknown. Thirdly, in light of previous research [2,3,4,5,6,7,8,9,10], metformin treatment may be particularly recommended to postmenopausal women with coexistent hypersecretion of other anterior pituitary hormones (subjects with hyperprolactinemia or primary hypothyroidism) resisting or poorly tolerating specific treatments (dopamine agonists and levothyroxine). Lastly, the putative anti-aging properties of metformin are probably absent in women with vitamin D insufficiency.

In the current study, a neutral effect on gonadotropin levels was observed in individuals with 25-hydroxyvitamin D levels between 20 and 30 ng/mL. For ethical reasons, no woman had 25-hydroxyvitamin D levels below 20 ng/mL. This finding suggests that even mild disturbances in vitamin D homeostasis may impair the impact of metformin on overactive gonadotropes. Considering the correlations between 25-hydroxyvitamin D levels and treatment-induced changes in gonadotropins and prolactin [26], it may be assumed that a deteriorating effect of hypovitaminosis D on the pituitary effects of metformin increases with its severity, which may be of importance in cases of patients with extremely high gonadotropin levels. Importantly, the impact of metformin on FSH and LH levels seems to be determined by actual vitamin D status. This explains why there were no differences in the response to metformin between two groups of women with 25-hydroxyvitamin D levels within the reference range. The drug decreased FSH and tended to reduce LH to the same degree in untreated women and in women receiving exogenous calciferol because of previous vitamin D deficiency (25-hydroxyvitamin D levels below 20 ng/mL [41]). Our findings cannot be explained by pharmacokinetic and/or pharmacodynamic interactions between exogenous metformin and calciferol contained in tablets because metformin action on FSH and LH did not correlate with the cumulative dose of exogenous vitamin D and the duration of calciferol supplementation. Thus, our study suggests that the pituitary effects of metformin in individuals with low vitamin D status may be restored by effective calciferol supplementation.

In line with our previous studies, metformin did not affect ovarian function in postmenopausal women, even in individuals with normal vitamin D status. In all groups of postmenopausal women, estradiol levels did not exceed the threshold value and did not change throughout the study. Moreover, the treatment had a neutral effect on AMH produced by the granulosa cells of preantral and small antral follicles and regarded as the most sensitive marker of ovarian reserve [42]. A dramatic decrease in the number of granulosa cells associated with menopause [43] explains why our findings are in contrast with a decrease in estradiol and progesterone secretion by isolated rat granulosa cells exposed to this drug [44]. A neutral effect on plasma estradiol is also in disagreement with a stimulatory effect of metformin on peripheral aromatization [45], the major source of estradiol in women after menopause [46]. Although we did not measure plasma metformin, the circulating levels and tissue content of this drug after long-term high-dose metformin treatment [47] were lower than the concentrations used in vitro studies [45]. Lastly, there were no correlations between the impact of metformin on gonadotropins and on estradiol and FSH.

Although metformin improved glucose homeostasis in all groups of postmenopausal women, the effect on glucose, insulin, HOMA1-IR, and HbA_1c_ was more pronounced in individuals with normal vitamin D status than in subjects with vitamin D insufficiency. The same relationships were observed previously in young women with elevated prolactin levels [26]. Thus, hypovitaminosis D may impair the metabolic effects of metformin in various age groups. Considering the association between glucose homeostasis disorders and vitamin D deficiency/insufficiency [19,20], as well as our findings, it seems reasonable to determine vitamin D status at least in individuals with an unexplained poor response to metformin but maybe also in other groups of subjects receiving metformin. Moreover, finding abnormally low 25-hydroxyvitamin D levels should justify calciferol supplementation. There are some possible explanations for our findings. Because elevated prolactin levels are a risk factor for insulin resistance and glucose abnormalities [48], a stronger effect of metformin on glucose homeostasis markers in individuals with prolactin excess [26] may be explained by differences in post-treatment prolactin levels. In the present study, they may be associated with putative unfavorable metabolic effects of elevated FSH levels [33,34]. Other authors suggest, however, that vitamin D may enhance the hypoglycemic properties of metformin by increasing insulin sensitivity in peripheral tissues, particularly in skeletal muscles [49]. Metformin and vitamin D may interact both at the level of the AMPK pathway in peripheral tissues and/or at the level of the glucose transporter type 4, mediating the rate-limiting glucose cellular uptake in adipocytes and muscle cells [50]. The activity of this transporter is up-regulated by both metformin [16,50] and calcitriol [51].

Contrary to previous studies suggesting seasonal variations in circulating levels of the investigated hormones in untreated subjects [29,30,31], there were no differences in metformin action between patients recruited in the winter and followed up in the summer months and patients recruited in the summer and followed up in the winter months. However, because of a small number of patients in each subgroup, our study might have been underpowered to detect relevant differences in metformin action. Thus, our findings do not exclude an association between the study outcomes and the period of the year when metformin was administered, and the question of whether the pituitary effects of this drug are season-dependent requires further research.

The mechanisms responsible for between-group differences in metformin action on plasma gonadotropins remain speculative. Because of rigorous inclusion criteria and the selection procedure, the study groups were well matched and differed only in 25-hydroxyvitamin D levels. Thus, our findings seem to be a consequence of differences in the vitamin D status of the patients. In line with this explanation, the degree of reduction in gonadotropin levels positively correlated with 25-hydroxyvitamin D levels. Because these levels remained constant over the entire study period, metformin does not seem to have had an impact on vitamin D homeostasis. This conclusion is in line with the findings of other research group [52] who reported a neutral effect of metformin on calciferol metabolism. Moreover, the lack of effect on estradiol and AMH as well as the lack of correlations between the impact on gonadotropins and on these hormones indicate that metformin action cannot be explained by the improvement in hypothalamic–pituitary–gonadal axis activity at the level of ovarian hormonal function (increased sensitivity of ovaries to gonadotropins or a direct stimulatory effect on ovarian steroidogenesis). Thus, the most likely explanation is that metformin and calciferol interact at the level of AMPK in gonadotropes. Although speculative, this explanation is supported by evidence from past studies. AMPK has been detected in high quantities in these cells [14]. Vitamin D insufficiency was found to down-regulate the AMPK pathway [53]. Lastly, AMPK mediated interactions between metformin and vitamin D in other tissues [24]. Metabolic factors do not play an important role in the regulation of FSH secretion (and probably also LH secretion) [54]. However, treatment-induced changes in FSH and LH levels correlated with the improvement in insulin sensitivity. This may be explained by simultaneous interactions between metformin and vitamin D at the level of various tissues: gonadotropin-secreting cells, adipocytes, and muscle cells.

Some study limitations merit consideration. Because of the small sample size, our findings do not allow us to draw definitive conclusions. Although vitamin D status was determined based on 25-hydroxyvitamin D levels, reflecting the free fractions of vitamin D metabolites [41], low 25-hydroxyvitamin D levels may be a result of decreased production or enhanced degradation. Because all individuals with 25-hydroxyvitamin D levels below 20 ng/mL should be supplemented with exogenous vitamin D preparations [55], our study excluded vitamin-D-deficient subjects. However, it would be interesting to assess the pituitary effects of metformin in this group of patients. The study does not provide insight into cellular and molecular aspects of the interactions between metformin and calciferol. It is uncertain whether the association with vitamin D status is the same in subjects with normal glucose homeostasis, not included in the current study. Lastly, although the study design minimized the impact of random diurnal, seasonal, and analytical variations in the assessed variables on the obtained results, we cannot totally exclude a potential impact on the obtained results of the regression-toward-the-mean effect [56].

## 5. Conclusions

Metformin reduced FSH levels and tended to reduce LH levels in postmenopausal women with normal vitamin D status but not in women with vitamin D insufficiency. The impact of this agent on concentrations of gonadotropins depended on their baseline secretion, 25-hydroxyvitamin D levels, and the degree of improvement in insulin sensitivity. The obtained results suggest that the impact of metformin on the gonadotrope secretory function in postmenopausal women is determined by the vitamin D status of patients. Because our study was a single-center prospective matched cohort study with a small sample size, the obtained results need to be confirmed in large-scale, well-designed clinical trials.

## Figures and Tables

**Table 1:** Baseline characteristics of participants.

Variable	Group A	Group B	Group C
**Number** (n)	30	29	29
**Age** (years)	59 ± 6	60 ± 6	60 ± 5
**Smokers** (%)	40	34	38
**Number of cigarettes a day** (n)	10 ± 5	11 ± 6	10 ± 6
**Duration of smoking** (years)	28 ± 12	30 ± 15	31 ± 14
**BMI** (kg/m^2^)	32.4 ± 5.2	32.2 ± 5.0	31.9 ± 4.9
**Systolic blood pressure** (mmHg)	136 ± 12	135 ± 13	134 ± 15
**Diastolic blood pressure** (mmHg)	87 ± 7	87 ± 6	87 ± 6

Group A: postmenopausal women with untreated vitamin D insufficiency. Group B: vitamin D preparation-naïve postmenopausal women with normal vitamin D status. Group C: vitamin-D-treated postmenopausal women with normal vitamin D status. Unless otherwise stated, the data are presented as the mean ± standard deviation. Abbreviations: BMI—body mass index.

**Table 2:** The impact of metformin on the investigated variables in metformin-treated postmenopausal women at high risk for diabetes.

Variable	Group A	Group B	Group C
**Glucose** (mg/dL)			
*Baseline*	112 ± 7	111 ± 7	110 ± 7
*Follow-up*	102 ± 8 *	97 ± 8 *^#^	96 ± 8 *^#^
**Insulin** (mU/L)			
*Baseline*	18.0 ± 4.2	17.5 ± 4.8	17.3 ± 3.9
*Follow-up*	12.9 ± 3.8 *	10.2 ± 2.8 *^#^	10.0 ± 3.1 *^#^
**HOMA1-IR**			
*Baseline*	5.0 ± 1.5	4.8 ± 1.4	4.7 ± 1.8
*Follow-up*	3.2 ± 1.1 *	2.4 ± 0.9 *^#^	2.4 ± 1.0 *^#^
**HbA_1c_** (%)			
*Baseline*	6.12 ± 0.16	6.10 ± 0.18	6.08 ± 0.20
*Follow-up*	5.85 ± 0.22 *	5.52 ± 0.24 *^#^	5.48 ± 0.26 *^#^
**25-hydroxyvitamin D** (ng/mL)			
*Baseline*	24.8 ± 2.5	42.9 ± 7.5 *	43.8 ± 7.6 *
*Follow-up*	25.1 ± 2.4	43.4 ± 7.1 *	44.6 ± 6.9 *
**FSH** (U/L)			
*Baseline*	68 ± 28	65 ± 23	62 ± 28
*Follow-up*	58 ± 19	43 ± 12 *^#^	42 ± 15 *^#^
**LH** (U/L)			
*Baseline*	42 ± 14	40 ± 13	41 ± 12
*Follow-up*	41 ± 11	34 ± 12 *^$^	35 ± 11 *^&^
**TSH** (mIU/L)			
*Baseline*	2.5 ± 1.0	2.3 ± 0.8	2.5 ± 0.9
*Follow-up*	2.3 ± 0.9	2.2 ± 1.0	2.4 ± 0.8
**Prolactin** (ng/mL)			
*Baseline*	14.0 ± 6.2	15.1 ± 5.0	14.9 ± 6.5
*Follow-up*	13.4 ± 5.5	14.8 ± 6.0	14.4 ± 6.7
**ACTH** (pg/mL)			
*Baseline*	42 ± 13	44 ± 16	39 ± 15
*Follow-up*	38 ± 18	41 ± 14	37 ± 17
**Estradiol** (pg/mL)			
*Baseline*	17 ± 5	18 ± 6	16 ± 7
*Follow-up*	18 ± 6	17 ± 6	16 ± 5
**Free thyroxine** (pmol/L)			
*Baseline*	16.0 ± 3.4	15.8 ± 3.2	15.6 ± 3.8
*Follow-up*	16.2 ± 3.9	15.5 ± 3.0	15.4 ± 3.5
**Free triiodothyronine** (pmol/L)			
*Baseline*	4.0 ± 1.2	3.9 ± 1.1	4.2 ± 1.3
*Follow-up*	3.8 ± 1.3	3.9 ± 1.2	4.1 ± 1.2
**IGF-1** (ng/mL)			
*Baseline*	110 ± 28	120 ± 32	124 ± 38
*Follow-up*	115 ± 30	118 ± 29	120 ± 31
**AMH** (pmol/L) ^1^			
*Baseline*	0.55 ± 0.65	0.52 ± 0.70	0.58 ± 0.64
*Follow-up*	0.52 ± 0.61	0.53 ± 0.62	0.60 ± 0.59

Group A: postmenopausal women with untreated vitamin D insufficiency. Group B: vitamin D preparation-naïve postmenopausal women with normal vitamin D status. Group C: vitamin-D-treated postmenopausal women with normal vitamin D status. The data are presented as the mean ± standard deviation. ^1^ Analysis of samples of 10 women from each group. * *p* < 0.05 vs. group A. ^#^ *p* < 0.05 vs. baseline value. ^$^ *p* = 0.0731 vs. baseline value. ^&^ *p* < 0.0521 vs. baseline value. Reference values for postmenopausal women: glucose: 70–99 mg/dL; insulin: 3–25 mIU/L; HOMA1-IR: <2.0; HbA_1c_: <5.6%; 25-hydroxyvitamin D: 30–60 ng/mL; FSH: >30 U/L; LH: >15 U/L; TSH: 0.4–4.5 mU/L; prolactin: 5.0–25.0 ng/mL; ACTH: 15–70 pg/mL; estradiol: <30 pg/mL; free thyroxine: 10.1–21.2 pmol/L; free triiodothyronine: 2.3–6.5 pmol/L; IGF-1: 70–165 ng/mL; AMH: <2.5 pmol/L. Reference values represent normal ranges for the laboratory that carried out the testing. Abbreviations: ACTH—adrenocorticotropic hormone; AMH—anti-Müllerian hormone; FSH—follicle-stimulating hormone; HbA_1c_—glycated hemoglobin; HOMA1-IR—the homeostatic model assessment 1 of insulin resistance ratio; IGF-1—insulin-like growth factor-1; LH—luteinizing hormone; TSH—thyroid-stimulating hormone.

**Table 3:** Percentage changes from baseline in metformin-treated postmenopausal women at high risk for diabetes.

Variable	Group A	Group B	Group C
**Δ Glucose**	−9 ± 5	−13 ± 6 *	−13 ± 7 *
**Δ** **Insulin**	−28 ± 16	−41 ± 18 *	−42 ± 20 *
**Δ HOMA1-IR**	−36 ± 20	−50 ± 26 *	−49 ± 23 *
**Δ HbA_1c_**	−4 ± 4	−10 ± 8 *	−10 ± 10 *
**Δ 25-hydroxyvitamin D**	1 ± 5	1 ± 7	2 ± 8
**Δ FSH**	−15 ± 15	−34 ± 18 *	−32 ± 16 *
**Δ LH**	−2 ± 8	−15 ± 12 *	−15 ± 16 *
**Δ TSH**	−8 ± 11	−4 ± 10	−4 ± 15
**Δ Prolactin**	−4 ± 8	−2 ± 10	−3 ± 8
**Δ ACTH**	−10 ± 16	−7 ± 14	−5 ± 12
**Δ Estradiol**	6 ± 26	−5 ± 28	0 ± 12
**Δ Free thyroxine**	1 ± 10	−1 ± 8	−1 ± 7
**Δ Free triiodothyronine**	−5 ± 16	0 ± 9	−2 ± 12
**Δ IGF-1**	4 ± 11	−2 ± 10	−3 ± 9
**Δ AMH**	−5 ± 24	2 ± 15	3 ± 18

Group A: postmenopausal women with untreated vitamin D insufficiency. Group B: vitamin D preparation-naïve postmenopausal women with normal vitamin D status. Group C: vitamin-D-treated postmenopausal women with normal vitamin D status. The data are presented as the mean ± standard deviation. * *p* < 0.05 vs. group A. Abbreviations: ACTH—adrenocorticotropic hormone; AMH—anti-Müllerian hormone; FSH—follicle-stimulating hormone; HbA_1c_—glycated hemoglobin; HOMA1-IR—the homeostatic model assessment 1 of insulin resistance ratio; IGF-1—insulin-like growth factor-1; LH—luteinizing hormone; TSH—thyroid-stimulating hormone.

**Table 4:** The impact of metformin on the assessed variables between patients recruited in the winter and summer months.

Variable	Patients Recruited in January and February	Patient Recruited in July or August
**Group A**		
**Δ Glucose**	−9 ± 7	−10 ± 8
**Δ** **Insulin**	−30 ± 20	−26 ± 18
**Δ HOMA1-IR**	−38 ± 24	−34 ± 25
**Δ HbA_1c_**	−4 ± 6	−4 ± 6
**Δ 25-hydroxyvitamin D**	2 ± 8	0 ± 8
**Δ FSH**	−16 ± 18	−14 ± 20
**Δ LH**	−2 ± 7	−3 ± 8
**Δ TSH**	−10 ± 14	−6 ± 13
**Δ Prolactin**	−3 ± 10	−4 ± 11
**Δ ACTH**	−13 ± 20	−8 ± 19
**Δ Estradiol**	7 ± 18	5 ± 16
**Δ Free thyroxine**	2 ± 8	1 ± 10
**Δ Free triiodothyronine**	−5 ± 18	−5 ± 19
**Δ IGF-1**	6 ± 17	2 ± 15
**Δ AMH**	−4 ± 30	−6 ± 20
**Group B**		
**Δ Glucose**	−13 ± 7	−14 ± 8
**Δ** **Insulin**	−45 ± 23	−37 ± 20
**Δ HOMA1-IR**	−53 ± 30	−47 ± 28
**Δ HbA_1c_**	−10 ± 9	−10 ± 9
**Δ 25-hydroxyvitamin D**	2 ± 12	−1 ± 11
**Δ FSH**	−32 ± 20	−36 ± 24
**Δ LH**	−15 ± 17	−15 ± 16
**Δ TSH**	−3 ± 12	−5 ± 15
**Δ Prolactin**	−2 ± 12	−3 ± 15
**Δ ACTH**	−6 ± 18	−9 ± 20
**Δ Estradiol**	−4 ± 30	−6 ± 32
**Δ Free thyroxine**	−1 ± 10	0 ± 8
**Δ Free triiodothyronine**	−2 ± 12	2 ± 12
**Δ IGF-1**	−1 ± 14	−4 ± 18
**Δ AMH**	4 ± 20	0 ± 22
**Group C**		
**Δ Glucose**	−14 ± 8	−13 ± 8
**Δ** **Insulin**	−44 ± 22	−41 ± 24
**Δ HOMA1-IR**	−51 ± 27	−48 ± 26
**Δ HbA_1c_**	−11 ± 12	−10 ± 11
**Δ 25-hydroxyvitamin D**	0 ± 9	3 ± 12
**Δ FSH**	−34 ± 18	−31 ± 20
**Δ LH**	−16 ± 20	−14 ± 19
**Δ TSH**	−6 ± 18	−3 ± 20
**Δ Prolactin**	−4 ± 10	−2 ± 11
**Δ ACTH**	−3 ± 18	−5 ± 20
**Δ Estradiol**	2 ± 13	−2 ± 16
**Δ Free thyroxine**	−2 ± 10	0 ± 12
**Δ Free triiodothyronine**	−5 ± 15	1 ± 16
**Δ IGF-1**	−1 ± 15	−5 ± 14
**Δ AMH**	1 ± 13	5 ± 24

Group A: postmenopausal women with untreated vitamin D insufficiency. Group B: vitamin D preparation-naïve postmenopausal women with normal vitamin D status. Group C: vitamin-D-treated postmenopausal women with normal vitamin D status. The data are presented as the mean ± standard deviation. Abbreviations: ACTH—adrenocorticotropic hormone; AMH—anti-Müllerian hormone; FSH—follicle-stimulating hormone; HbA_1c_—glycated hemoglobin; HOMA1-IR—the homeostatic model assessment 1 of insulin resistance ratio; IGF-1—insulin-like growth factor-1; LH—luteinizing hormone; TSH—thyroid-stimulating hormone.

## Data Availability

The data that support the findings of this study are available from the corresponding author upon reasonable request.

## Abbreviations

ACTH—adrenocorticotropic hormone; AMH—anti-Müllerian hormone; AMPK—5′-adenosine monophosphate-activated protein kinase; BMI—body mass index; FSH—follicle-stimulating hormone; HbA1c—glycated hemoglobin, HOMA1-IR—the homeostatic model assessment 1 of insulin resistance ratio; IGF-1—insulin-like growth factor-1; LH—luteinizing hormone; TSH—thyroid-stimulating hormone

## References

[1] Zhou J., Massey S., Story D., Li L. (2018). Metformin: An old drug with new applications. Int. J. Mol. Sci..

[2] Cappelli C., Rotondi M., Pirola I., Agosti B., Gandossi E., Valentini U., de Martino E., Cimino A., Chiovato L., Agabiti-Rosei E. (2009). TSH-lowering effect of metformin in type 2 diabetic patients: Differences between euthyroid, untreated hypothyroid, and euthyroid on L-T4 therapy patients. Diabetes Care.

[3] Lupoli R., Di Minno A., Tortora A., Ambrosino P., Lupoli G.A., Di Minno M.N. (2014). Effects of treatment with metformin on TSH levels: A meta-analysis of literature studies. J. Clin. Endocrinol. Metab..

[4] Bo Q.J., Wang Z.M., Li X.B., Ma X., Wang C.Y., de Leon J. (2016). Adjunctive metformin for antipsychotic-induced hyperprolactinemia: A systematic review. Psychiatry Res..

[5] Krysiak R., Kowalcze K., Szkróbka W., Okopień B. (2016). The effect of metformin on prolactin levels in patients with drug-induced hyperprolactinemia. Eur. J. Intern. Med..

[6] Krysiak R., Szkróbka W., Okopień B. (2018). The effect of metformin on serum gonadotropin levels in postmenopausal women with diabetes and prediabetes: A pilot study. Exp. Clin. Endocrinol. Diabetes.

[7] Krysiak R., Kowalcze K., Okopień B. (2021). The impact of metformin on prolactin levels in postmenopausal women. J. Clin. Pharm. Ther..

[8] Krysiak R., Szkróbka W., Bednarska-Czerwińska A., Okopień B. (2021). Plasma gonadotropin levels in metformin-treated men with prediabetes: A non-randomized, uncontrolled pilot study. Fundam. Clin. Pharmacol..

[9] Jiang S., Tang T., Sheng Y., Li R., Xu H. (2022). The effects of letrozole and metformin combined with targeted nursing care on ovarian function, LH, and FSH in infertile patients with polycystic ovary syndrome. J. Healthc. Eng..

[10] Azizi Kutenaei M., Hosseini Teshnizi S., Ghaemmaghami P., Eini F., Roozbeh N. (2021). The effects of myo-inositol vs. metformin on the ovarian function in the polycystic ovary syndrome: A systematic review and meta-analysis. Eur. Rev. Med. Pharmacol. Sci..

[11] Krysiak R., Kowalcze K., Okopień B. (2023). Rosuvastatin potentiates gonadotropin-lowering effects of metformin in postmenopausal women: A pilot study. Pharmacology.

[12] Ueno M. (2007). Molecular anatomy of the brain endothelial barrier: An overview of the distributional features. Curr. Med. Chem..

[13] Labuzek K., Suchy D., Gabryel B., Bielecka A., Liber S., Okopień B. (2010). Quantification of metformin by the HPLC method in brain regions, cerebrospinal fluid and plasma of rats treated with lipopolysaccharide. Pharmacol. Rep..

[14] Tosca L., Froment P., Rame C., McNeilly J.R., McNeilly A.S., Maillard V., Dupont J. (2011). Metformin decreases GnRH- and activin-induced gonadotropin secretion in rat pituitary cells: Potential involvement of adenosine 5′ monophosphate-activated protein kinase (PRKA). Biol. Reprod..

[15] Aslam M., Ladilov Y. (2022). Emerging role of cAMP/AMPK signaling. Cells.

[16] Rena G., Hardie D.G., Pearson E.R. (2017). The mechanisms of action of metformin. Diabetologia.

[17] Tossetta G. (2022). Metformin improves ovarian cancer sensitivity to paclitaxel and platinumpbased drugs: A review of in vitro findings. Int. J. Mol. Sci..

[18] Jeong H.G., Park H. (2022). Metabolic disorders in menopause. Metabolites.

[19] Contreras-Bolívar V., García-Fontana B., García-Fontana C., Muñoz-Torres M. (2021). Mechanisms involved in the relationship between vitamin D and insulin resistance: Impact on clinical practice. Nutrients.

[20] Wimalawansa S.J. (2018). Associations of vitamin D with insulin resistance, obesity, type 2 diabetes, and metabolic syndrome. J. Steroid Biochem. Mol. Biol..

[21] Melguizo-Rodríguez L., Costela-Ruiz V.J., García-Recio E., De Luna-Bertos E., Ruiz C., Illescas-Montes R. (2021). Role of vitamin D in the metabolic syndrome. Nutrients.

[22] Lerchbaum E. (2014). Vitamin D and menopause—A narrative review. Maturitas.

[23] Khan D.M., Jamil A., Randhawa F.A., Butt N.F., Malik U. (2018). Efficacy of oral vitamin D on glycated haemoglobin (HbA1c) in type 2 diabetics having vitamin D deficiency—A randomized controlled trial. J. Pak. Med. Assoc..

[24] Chang E. (2022). Effects of vitamin D supplementation on adipose tissue inflammation and NF-κB/AMPK activation in obese mice fed a high-fat diet. Int. J. Mol. Sci..

[25] Li H.X., Gao J.M., Liang J.Q., Xi J.M., Fu M., Wu Y.J. (2015). Vitamin D3 potentiates the growth inhibitory effects of metformin in DU145 human prostate cancer cells mediated by AMPK/mTOR signalling pathway. Clin. Exp. Pharmacol. Physiol..

[26] Krysiak R., Kowalcze K., Okopień B. (2021). Vitamin D status determines the impact of metformin on circulating prolactin levels in premenopausal women. J. Clin. Pharm. Ther..

[27] Krysiak R., Kowalcze K., Okopień B. (2020). The impact of combination therapy with metformin and exogenous vitamin D on hypothalamic-pituitary-thyroid axis activity in women with autoimmune thyroiditis and high-normal thyrotropin levels. J. Clin. Pharm. Ther..

[28] Diabetes Prevention Program Research Group (2002). Reduction in the incidence of type 2 diabetes with lifestyle intervention or metformin. N. Engl. J. Med..

[29] Benucci A., Tommasi M., Fantappié B., Scardigli S., Ottanelli S., Pratesi E., Romano S. (1993). Serum 25-hydroxyvitamin D levels in normal subjects: Seasonal variations and relationships with parathyroid hormone and osteocalcin. J. Nucl. Biol. Med..

[30] Kivelä A., Kauppila A., Ylöstalo P., Vakkuri O., Leppäluoto J. (1988). Seasonal, menstrual and circadian secretions of melatonin, gonadotropins and prolactin in women. Acta Physiol. Scand..

[31] Kauppila A., Pakarinen A., Kirkinen P., Mäkilä U. (1987). The effect of season on the circulating concentrations of anterior pituitary, ovarian and adrenal cortex hormones and hormone binding proteins in the subarctic area; evidence of increased activity of the pituitary-ovarian axis in spring. Gynecol. Endocrinol..

[32] Morisky D.E., Green L.W., Levine D.M. (1986). Concurrent and predictive validity of a self-reported measure of medication adherence. Med. Care.

[33] Taneja C., Gera S., Kim S.M., Iqbal J., Yuen T., Zaidi M. (2019). FSH-metabolic circuitry and menopause. J. Mol. Endocrinol..

[34] Lizneva D., Rahimova A., Kim S.M., Atabiekov I., Javaid S., Alamoush B., Taneja C., Khan A., Sun L., Azziz R. (2019). FSH byond fertility. Front. Endocrinol..

[35] Takahashi T.A., Johnson K.M. (2015). Menopause. Med. Clin. N. Am..

[36] Blair J.A., Bhatta S., McGee H., Casadesus G. (2015). Luteinizing hormone: Evidence for direct action in the CNS. Horm. Behav..

[37] Barron A.M., Verdile G., Martins R.N. (2006). The role of gonadotropins in Alzheimer’s disease: Potential neurodegenerative mechanisms. Endocrine.

[38] Flores V.A., Pal L., Manson J.E. (2021). Hormone therapy in menopause: Concepts, controversies, and approach to treatment. Endocr. Rev..

[39] Lv Z., Guo Y. (2020). Metformin and its benefits for various diseases. Front. Endocrinol..

[40] Hui F., Zhang Y., Ren T., Li X., Zhao M., Zhao Q. (2019). Role of metformin in overweight and obese people without diabetes: A systematic review and network meta-analysis. Eur. J. Clin. Pharmacol..

[41] Holick M.F. (2009). Vitamin D status: Measurement, interpretation, and clinical application. Ann. Epidemiol..

[42] Di Clemente N., Racine C., Pierre A., Taieb J. (2021). Anti-Müllerian hormone in female reproduction. Endocr. Rev..

[43] Shifren J.L., Schiff I. (2000). The aging ovary. J. Womens Health Gend. Based Med..

[44] Tosca L., Solnais P., Ferré P., Foufelle F., Dupont J. (2006). Metformin-induced stimulation of adenosine 5′ monophosphate-activated protein kinase (PRKA) impairs progesterone secretion in rat granulosa cells. Biol. Reprod..

[45] Rice S., Pellatt L., Ramanathan K., Whitehead S.A., Mason H.D. (2009). Metformin inhibits aromatase via an extracellular signal-regulated kinase-mediated pathway. Endocrinology.

[46] Simpson E., Rubin G., Clyne C., Robertson K., O’Donnell L., Davis S., Jones M. (1999). Local estrogen biosynthesis in males and females. Endocr. Relat. Cancer.

[47] Graham G.G., Punt J., Arora M., Day R.O., Doogue M.P., Duong J.K., Furlong T.J., Greenfield J.R., Greenup L.C., Kirkpatrick C.M. (2011). Clinical pharmacokinetics of metformin. Clin. Pharmacokinet..

[48] Auriemma R.S., De Alcubierre D., Pirchio R., Pivonello R., Colao A. (2019). Glucose abnormalities associated to prolactin secreting pituitary adenomas. Front. Endocrinol..

[49] Amin S.N., Hussein U.K., Yassa H.D., Hassan S.S., Rashed L.A. (2018). Synergistic actions of vitamin D and metformin on skeletal muscles and insulin resistance of type 2 diabetic rats. J. Cell. Physiol..

[50] Huang S., Czech M.P. (2007). The GLUT4 glucose transporter. Cell Metab..

[51] Manna P., Achari A.E., Jain S.K. (2018). 1,25(OH)_2_-vitamin D_3_ upregulates glucose uptake mediated by SIRT1/IRS1/GLUT4 signaling cascade in C2C12 myotubes. Mol. Cell. Biochem..

[52] Out M., Top W.M., Lehert P., Schalkwijk C.A., Stehouwer C.D., Kooy A. (2018). Long-term treatment with metformin in type 2 diabetes and vitamin D levels: A post-hoc analysis of a randomized placebo-controlled trial. Diabetes Obes. Metab..

[53] Chang E., Kim Y. (2017). Vitamin D insufficiency exacerbates adipose tissue macrophage infiltration and decreases AMPK/SIRT1 activity in obese rats. Nutrients.

[54] Das N., Kumar T.R. (2018). Molecular regulation of follicle-stimulating hormone synthesis, secretion and action. J. Mol. Endocrinol..

[55] Haines S.T., Park S.K. (2012). Vitamin D supplementation: What’s known, what to do, and what’s needed. Pharmacotherapy.

[56] Barnett A.G., van der Pols J.C., Dobson A.J. (2005). Regression to the mean: What it is and how to deal with it. Int. J. Epidemiol..

